# First Trimester Uterine Rupture: A Case Report and Literature Review

**DOI:** 10.3390/ijerph17082976

**Published:** 2020-04-24

**Authors:** Fabiana Cecchini, Alice Tassi, Ambrogio P. Londero, Giovanni Baccarini, Lorenza Driul, Serena Xodo

**Affiliations:** Department of Gynecology and Obstetrics, School of Medicine of Udine, 33100 Udine, Italy; cecchinifabiana@gmail.com (F.C.); alicetassi@gmail.com (A.T.); ambrogio.londero@gmail.com (A.P.L.); baccag@hotmail.it (G.B.); lorenza.driul@uniud.it (L.D.)

**Keywords:** first trimester, uterine rupture, cesarean section

## Abstract

The aim is to report a case of spontaneous uterine rupture in the first trimester of pregnancy and to review the literature on the topic. Methods: A literature search was performed using PubMed and Scopus. Relevant English articles were identified without any time or study limitations. The data were aggregated, and a summary statistic was calculated. Results: A 35-year-old gravida 5, para 2 was admitted at our department because of fainting and abdominal pain. The woman had a first-trimester twin pregnancy and a history of two previous cesarean sections (CSs). Suspecting a uterine rupture, an emergency laparotomy was performed. The two sacs were completely removed, and the uterine rupture site was closed with a double-layer suture. The patient was discharged from hospital four days later in good condition. On the basis of this experience, a total of 76 case reports were extracted from PubMed and included in the review. Fifty-three patients out of 76 (69.74%) underwent previous surgery on the uterus. Most women (67.92%) had a CS, and in this group a cesarean scar pregnancy (CSP) or a placenta accreta spectrum (PAS) disorder was found to be the etiology in 77.78% of cases. Furthermore, 35.85% of the women had hysterectomy after uterine rupture. Twenty-three patients out of 76 (30.26%) had an unscarred uterus. Of this group, most women presented a uterine anomaly (43.48%). Moreover, 17.39% of these women had a hysterectomy. Conclusion: According to the literature, the current pandemic use of CS explains most cases of first-trimester uterine rupture.

## 1. Introduction

Rupture of the uterus is a rare, serious, and potentially fatal complication of pregnancy. A uterine rupture is defined as a disruption of the whole uterine muscle and the visceral peritoneum. This term is often confused with uterine dehiscence, in which the integrity of the visceral peritoneum is retained [1].

Most uterine ruptures occur at the term of pregnancy and only a limited number of cases occur in the first or second trimester of pregnancy [2]. The main risk factor for uterine rupture is previous surgery with opening of the endometrial cavity. There are however cases of uterine rupture where the cause remains unknown. Our aim is to review the literature after having presented a case report of spontaneous uterine rupture in the first trimester of pregnancy. The specific objectives of the systematic review expressed in terms of population, exposure, and outcome are reported in Supplementary Table S1.

## 2. Materials and Methods 

**Figure 1:**
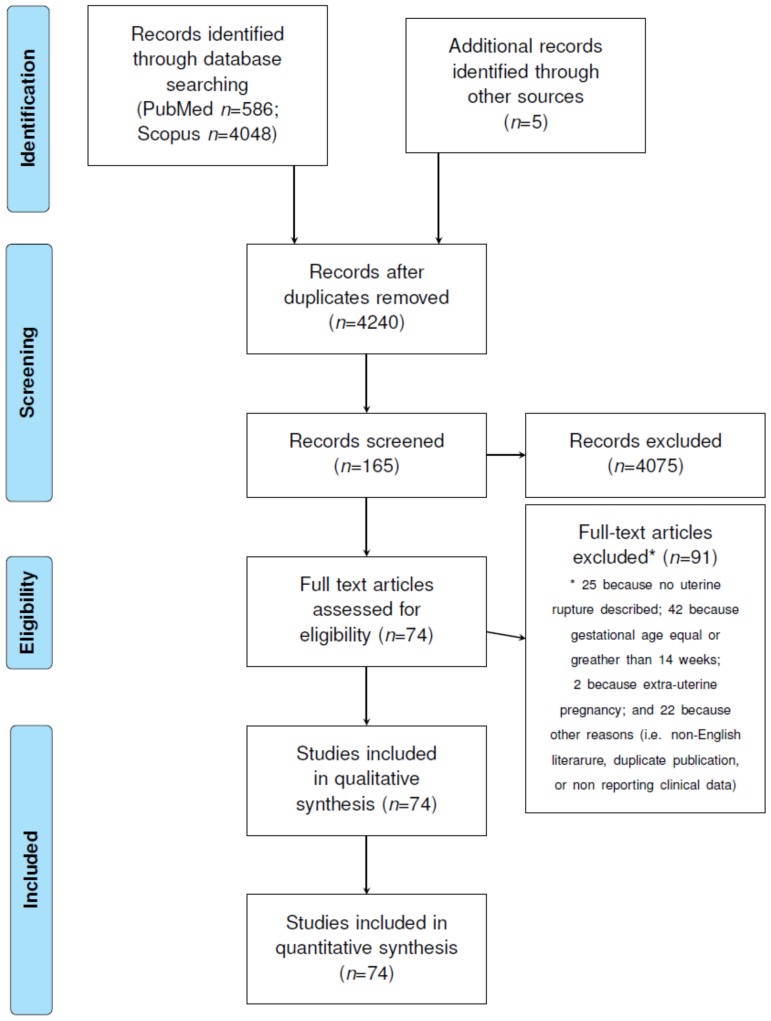
Flow chart of the study based on a Preferred Reporting Items for Systematic Reviews and Meta-Analyses (PRISMA) statement.

A search literature was last time performed on 10th April 2020 using Scopus (www.scopus.com, accessed on 10.04.2020) and PubMed (www.ncbi.nlm.nih.gov, accessed on 10.04.2020) databases. Articles describing one or more cases of uterine rupture in the first trimester were identified from the above databases without any time or study limitations but considering only articles in English language. The search terms were “first trimester AND uterine rupture”, “early rupture AND gravid uterus”, “rupture of the uterus in the first trimester”, “pregnancy hemoperitoneum AND placenta percreta”, “cesarean scar AND uterine rupture AND first trimester”, “extrauterine pregnancy AND early uterine rupture”. The specific queries are presented in Supplementary Table S2. All non-duplicate, identified articles were independently reviewed by two authors (F.C. and S.X.), and relevant scientific articles were selected by mutual agreement. Studies were deemed eligible for inclusion in the present review if they described at least one case of first-trimester uterine rupture. The whole process is described in Figure 1.

Supplementary List S1 shows the included and excluded full-text articles and the reason for exclusion. The following information was collected from the case reports: age, parity, maternal comorbidity, type of previous uterine surgery, previous uterine rupture, singleton or twin pregnancy, assisted reproductive technology (ART) or spontaneous pregnancy, uterotonics use, the presence of uterine anomaly, evidence of cesarean scar pregnancy (CSP), or placenta accreta spectrum (PAS) disorder. The uterine anomalies considered in the case reports were bicornuate uterus with or without rudimentary horn, and T-shaped uterus. As for the clinical picture, cases presenting shock with stable vitals were differentiated from cases with life-threatening shock. As for the type of intervention, cases where the uterine rupture was repaired were distinguished from those cases that unfortunately ended in hysterectomy; the type of abdominal approach (laparotomy vs. laparoscopy), the amount of blood loss, and the results of histological examination were also collected. 

**Figure 2:**
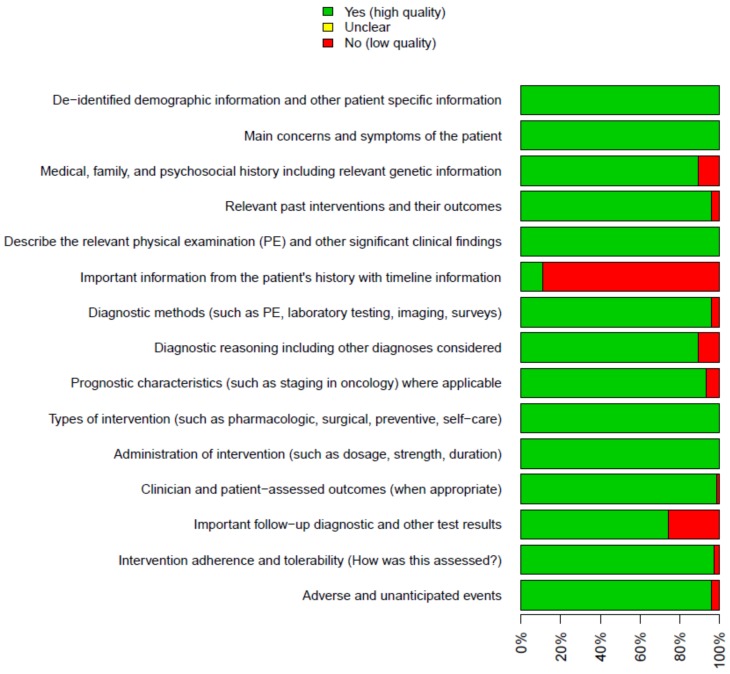
Methodological quality summary, considering all included studies in this meta-analysis and each specific methodological quality item.

The Preferred Reporting Items for Systematic Reviews and Meta-Analyses (PRISMA) guidelines were used to prepare this manuscript (Figure 1) [3]. The literature quality has been evaluated through the use of the CARE (Case Report) check list. In particular, the presence of the following items was assessed: patient information, clinical findings, the presence of timeline information, diagnostic assessment, therapeutic intervention, and outcomes [4,5]. The presence (+) of information concerning a specific item was considered to be a sign of a study with a low risk of bias, while the absence (-) of an item was considered to be indicative of a high risk of bias. In some cases, the item was classified as unclear (?), due to the absence of sufficient information (Figure 2, Supplementary Figures S1 and S2).

### 2.1. Statistical Analysis

The analysis was performed using R (version 3.6.0; R: A language and environment for statistical computing. R Foundation for Statistical Computing, Vienna, Austria). Data from case reports were aggregated and a summary statistic was calculated. The risk-of-bias assessment was not possible for this study. This case report and case series meta-analysis is exempt from ethical approval as the analysis involves only already published and anonymized data.

### 2.2. Case Report

A 35-year-old gravida 5, para 2 (1122) was admitted to the emergency room because of fainting and abdominal pain. The woman was at 11 weeks and 4 days of gestation and had a twin pregnancy complicated by hypertension on therapy. She had a history of two previous cesarean sections (CS). The first one was performed in 2015 for non-reassuring fetal status at 38 weeks of gestation; the second one in 2017 because of preeclampsia and ascites at 32 weeks. During the second cesarean section, fetal extraction was difficult, therefore a T incision had been necessary.

At admission, the woman had hypotension (her blood pressure was 90/60 mmHg), a cardiac frequency of 78 bpm, and anemia (her hemoglobin was 9.7 g/dL). On abdominal examination, there was generalized tenderness. The transvaginal ultrasound scan showed an intrauterine twin pregnancy with two gestational sacs and two alive fetuses with a crown–rump length of 46 and 47 mm, respectively. The scan also demonstrated the presence of free fluid in the abdomen. Based on these clinical, laboratory, and sonographic findings, a uterine rupture was suspected. Once proper consent was obtained, an emergency laparotomy was performed. The procedure confirmed a hemorrhage in the peritoneal cavity. After clot removal, a defect appeared in the lower anterior uterine wall, through which placental tissue and part of the gestational sac could be directly observed. With gentle squeezing the two sacs were completely removed undamaged from the uterine wall, and then the rupture site was closed with a double-layer suture. The total blood loss during the surgical procedure was 2000 mL. One unit of packed red cells (PRCs) was infused during surgery. The patient suffered no side-effects and was discharged from hospital four days later in good condition. The histological examination showed two embryos, consistent for 11 weeks of gestation, of female gender still inside the amniotic sac, which remained surprisingly intact. The examination of the main parenchymal viscera did not produce significant findings. Histological examination of the placentas showed placental parenchyma with villous maturation consistent with gestational age, with normal cotyledonal branching, as well as focal involutional villous fibro-edematous alterations and hemorrhagic areas suggestive for focal retroplacental hemorrhage.

### 2.3. Systematic Review of the Literature

A total of 74 [6,7,8,9,10,11,12,13,14,15,16,17,18,19,20,21,22,23,24,25,26,27,28,29,30,31,32,33,34,35,36,37,38,39,40,41,42,43,44,45,46,47,48,49,50,51,52,53,54,55,56,57,58,59,60,61,62,63,64,65,66,67,68,69,70,71,72,73,74,75,76,77,78,79] papers describing 76 case reports were included in the review (Figure 1). Most of studies were of high quality (Figure 2, Supplementary Figures S1 and S2).

The median maternal age was 32 years (IQR 27–34), and 10 women were nulliparous (26.32%) (Table 1).

In total, 69.74% of the cases (53/76) underwent previous surgery on their uterus. Thirty-six women out of 53 underwent a CS (36/53, 67.92%), fifteen women had dilation and curettage (15/53, 28.30%), five women had myomectomy (5/53, 9.43%), and two other women had cornual resection (2/53, 3.77%). By comparing women with an unscarred uterus to women with previous uterine surgery, Table 2 shows the factors associated with first-trimester uterine rupture and the histological findings.

Considering the subgroup of women with CS in their obstetric history, most women had a cesarean scar pregnancy (CSP) or a placenta accreta spectrum (PAS) disorder diagnosed (28/36, 77.78%). In more detail: twenty-four (24/36, 66.67%) had a diagnosis of CSP, thirteen (13/36, 36.11%) had a diagnosis of PAS disorder, while eight (9/36, 25.00%) reported both conditions. Of the remaining 8 women, 4 underwent a termination of pregnancy (TOP) or medical evacuation of spontaneous miscarriage induced with drugs (4/36, 11.11%) [28,33,36,37], and, in the other 4 cases, it was impossible to recognize specific triggers for first-trimester uterine rupture (4/36, 11.11%) [47,60,67,74].

Among women with previous dilation and curettage, 4 (4/15, 26.67%) also had a CS in their past [14,19,47,73], 5 had a diagnosis of PAS disorder (5/15, 33.33%) [19,21,23,46,73], 1 had a TOP with drugs (1/15, 8.33%) [38], 1 had a previous uterine rupture in the past (1/15, 8.33%) [50], and in 2 cases no predisposing factors for uterine rupture were clearly identified (16.67%) [15,27]. 

In the group with scarred uteri, 5 women underwent myomectomy in their gynecological history [43,51,52,62,72]. Looking for trigger factors for uterine rupture after this surgical procedure, it was found that 2/5 (40%) had a diagnosis of CSP [62,72], 2/5 (40%) had a pregnancy obtained with ART [51,72], and a further 2 cases (2/5, 40%) remained unexplained [43,52].

As for women with cornual resection in the past [13]: one had endometriosis, while the other case had a PAS disorder and was a twin pregnancy. Both were ART-induced pregnancies. 

**Figure 3:**
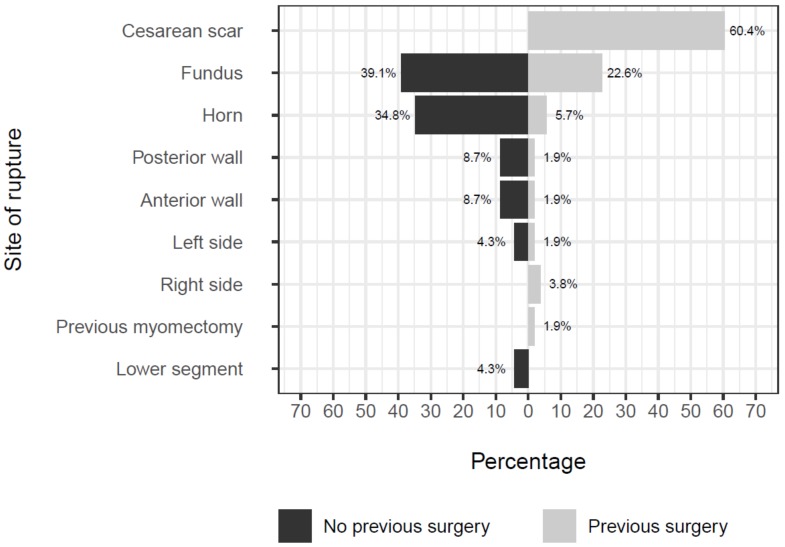
Plot showing rupture site among patients with and without previous surgery.

Figure 3 is helpful to understand the whole picture from the perspective of the rupture site. Interestingly, most of uterine ruptures in women with previous surgery occurred at the site of the cesarean scar. On the other hand, most of the ruptures in unscarred uteri happened in the fundus or in the horn.

The 35.85% (19/53) of women with previous surgery had a hysterectomy after their first-trimester uterine rupture. In 33 cases (33/53, 62.26%), the clinical presentation picture was dramatic with life-threatening shock; of those, 14 (14/33, 42.42%) underwent hysterectomy. 

Out of 76 patients, 23 (23/76, 30.26%) did not have any previous uterine surgery, presenting therefore an unscarred uterus (Table 1). Of this group, most cases had a uterine anomaly (10/23, 43.48%), 3 had a PAS disorder (3/23, 13.04%), 2 had an ART-induced pregnancy (2/23, 8.70%), 2 had a pharmacological termination of pregnancy (2/23, 8.70%), 3 others were multiparous (three or more previous births) (3/23, 13.04%) [9,16,26] (Table 1, Table 2, and Supplementary Tables S3–S5). However, in 4 cases, no clearly recognizable predisposing factor was present (4/19, 21.05%) [8,12,55,77].

Four women out of 23 had a hysterectomy (4/23, 17.39%), while 12 had a life- threatening shock (12/23, 42.17%) (Table 1).

## 3. Discussion

This systematic review summarizes all cases of first-trimester uterine rupture reported in the literature. It demonstrates that most first-trimester uterine ruptures occur in a scarred uterus (69.74%), and the top three surgical interventions associated for the uterine wall disruption are cesarean section (67.92%), dilation and curettage (28.30%), and myomectomy (9.43%). However, first-trimester uterine rupture may happen in unscarred uteri as well. This systematic review shows that a strong association exists between first trimester unscarred uterine rupture and uterine anomaly (43.48%), and a minor correlation exists with multiparity (17.39%). 

### 3.1. First-Trimester Uterine Rupture in Scarred Uterus

A uterine scar may range from a small defect of the decidua and superficial myometrium to a wide and deep defect involving all the myometrium from the endometrial cavity to the uterine serosa [80]. According to recent evidence, the PAS disorder is mainly attributed to surgical damage, which impairs the normal decidualization disrupting the integrity of the uterine endometrium and myometrium [81]. Human placentation is highly invasive. After implantation, cytotrophoblast cells proliferate in columns that merge surrounding the conceptus. These cells on the outer surface, forming the extra-villous trophoblast (EVT), invade the decidual stroma up to the inner third of the myometrium. Invasion of the uterine tissue beyond this level is likely determined by the absence of endometrium in the scar area, rather than by an uncontrolled invasion of the EVT [81]. Therefore, lacking the decidua that regulates the placenta adhesion to the uterine wall, the scar area seems to be particularly attractive for the growing placenta. According to this new perspective, the PAS disorder is primarily due to the dehiscence of the scar, which allows the trophoblast to penetrate unopposed into the myometrium [82]. Due to its pandemic rate increase, cesarean delivery has become the major driver of all grades of accreta placentation. However, smaller and more superficial damage to the uterine wall determined by minor procedures such as curettage and manual removal of placenta, may also be responsible for morbidly adherent placentation. Of note, one prior cesarean delivery, especially if prelabor, is sufficient to increase the occurrence of PAS disorders in subsequent pregnancy [83]. Why a CS performed before the onset of labor determines a higher risk of abnormal placentation is still unclear. It is possible that uterine tissue gets thinner during labor, thus minimizing the amount of damage determined by the incision. Alternatively, it might be speculated that the cesarean incision performed after the onset of labor usually takes place next to the cervix rather than in the lower uterine segment, which tends more easily to develop an abnormal placentation in future pregnancy.

When the blastocyst implants on a cesarean scar or in its niche, a CSP may develop. The niche usually appears as a discontinuity of the uterine wall in the site of the scar and is the result of an abnormal healing of a previous cesarean incision. In case of CSP in the niche, no measurable myometrial thickness separates the gestational sac from the bladder, according to an ultrasound study [82]. Recent evidence has demonstrated, however, that CSP is an early form of PAS disorder [82,84]. In the absence of a voluntary termination of pregnancy, a CSP, if left continuing, may undergo a spontaneous demise or not [85]. After 7 weeks, the gestational sac moves toward the fundus resulting in an intrauterine pregnancy but leaving behind the placenta and its important vascularization. If not timely recognized, this CSP could be misdiagnosed as a normal intrauterine pregnancy instead of a CSP evolving in a pregnancy affected by placental disorder, with dramatically adverse outcomes for both the mother and the fetus. The commonly recognized complications are vaginal or intra-abdominal bleeding, uterine rupture, and shock, occurring in the second and third trimester and requiring a lifesaving surgical intervention. Our systematic review demonstrates that CSP could lead to a first-trimester uterine rupture, which is up to now often unpredictable and potentially devastating. In our opinion, clinicians should be aware of this eventuality when they counsel women carrying a CSP. Historically, counseling of this particular issue has changed from advising women to terminate pregnancy implanted on the scar to informing them that continuing pregnancy could lead to live neonates despite the high risk of hysterectomy [82]. When opting for continuing pregnancy, the patient accepts the risk to undergo a demolitive but lifesaving intervention in late gestation. However, this lifesaving intervention might be necessary earlier, in a pre-viable period, if a uterine rupture with an uncontrolled hemorrhage occurs in the first trimester.

### 3.2. First-Trimester Uterine Rupture in Unscarred Uterus

This systematic review shows that first-trimester unscarred uterine rupture is mostly correlated with a specific uterine anomaly, i.e., bicornuate uterus. Uterine malformations are the result of disturbances occurring at different stages during the Mullerian ducts’ development in fetal life [86]. Depending on the exact point the interruption occurs, i.e., differentiation, migration, fusion, and canalization of the Mullerian ducts, there could be a spectrum of anomalies. At one extreme of this spectrum there is the Mullerian agenesis, characterized by failure of development of the ducts. At the other end of the spectrum, one can find arcuate uterus, which is a mild abnormality of canalization, identified by an indentation of the uterine fundus toward the cavity [86]. Bicornuate uterus is positioned between these two extremes and, being a unification defect, it derives from an abnormal fusion of the ducts. Literature data show that patients with a uterine anomaly have a poor pregnancy outcome, with a tendency to undergo abortion, preterm delivery, or operative birth [87,88]. The higher risk of rupture of anomalous uteri in the first trimester is still unexplained. According to the literature, bicornuate uteri show a peculiar vascular network between the 2 hemi-cavities, designing the Greek letter y at the level of the midline [89]. It could be hypothesized that this type of vascularization weakens the uterine wall, particularly at the level of the fundus, where the spontaneous rupture usually happens. Unscarred uterine rupture in the first trimester has been demonstrated to be associated with multiparity. However, it is difficult to explain this causal link in biological terms.

To our knowledge, this is the first systematic review on the topic. A systematic review offers the unique opportunity to analyze a rare event such as RUP, which otherwise would be difficult to explore thorough cohort studies, either retrospectively or prospectively. The main limitation is represented by the low prevalence of this rare pregnancy complication. Case studies can be prone to bias mostly for inconsistencies in the information provided or in the outcome definitions, thus limiting the generalizability to larger populations of patients.

## 4. Conclusions

Uterine rupture in the first trimester of pregnancy is a rare and life-threatening occurrence that can be safely and conservatively treated. The main driving factor behind a scarred uterus is previous CS. Therefore, a careful ultrasound examination is recommended in order to timely recognize the PAS disorder associated with previous uterine surgery, with the aim of an appropriate counseling and a better management of these cases. The main factor correlated with first-trimester uterine rupture in unscarred uteri is uterine anomaly, which, if known, deserves particular attention during the whole pregnancy.

## Figures and Tables

**Table 1:** Aggregated data of maternal features, previous surgery, and management of first-trimester uterine rupture cases from the systematic review of the literature.

Variables	All (76)	No Previous Surgery (23)	Previous Surgery (53)
Age (years)	32 (27–34)	29 (24–33)	33 (27–36)
Parity	13.16% (10/76)	30.43% (7/23)	5.66% (3/53)
Para 0	26.32% (20/76)	43.48% (10/23)	18.87% (10/53)
Para 1–2	53.95% (41/76)	39.13% (9/23)	60.38% (32/53)
Para ≥3	19.74% (15/76)	17.39% (4/23)	20.75% (11/53)
Comorbidities	21.05% (16/76)	8.70% (2/23)	26.42% (14/53)
Leiomyoma	5.26% (4/76)	0.00% (0/23)	7.55% (4/53)
Adenomyosis	3.95% (3/76)	4.35% (1/23)	3.77% (2/53)
Twins	6.58% (5/76)	8.70% (2/23)	5.66% (3/53)
Type of previous surgery			
CS			67.92% (36/53)
D&C			28.3% (15/53)
Myomectomy			9.43% (5/53)
Cornual resection			3.77% (2/53)
Months since previous surgery			24 (10–36)
Treatment			
Diagnostic intervention	39.47% (30/76)	47.83% (11/23)	35.85% (19/53)
Lifesaving surgery	59.21% (45/76)	52.17% (12/23)	62.26% (33/53)
LPS	9.21% (7/76)	13.04% (3/23)	7.55% (4/53)
LPT	75% (57/76)	78.26% (18/23)	73.58% (39/53)
Conversion from LPS to LPT	14.47% (11/76)	8.70% (2/23)	16.98% (9/53)
Defect repair	63.16% (48/76)	73.91% (17/23)	58.49% (31/53)
Hysterectomy	30.26% (23/76)	17.39% (4/23)	35.85% (19/53)
Total blood loss (mL)	1800 (1000–2500)	2250 (1500–2625)	1500 (1000–2000)

PS = previous surgery; D&C = dilation and curettage; CS = cesarean section; LPS = laparoscopy; LPT = laparotomy; NK = not known.

**Table 2:** Aggregated data considering associated factors with first-trimester uterine rupture and histological findings after surgical intervention.

Variables	All (76)	No Previous Surgery (23)	Previous Surgery (53)
ART induced	11.84% (9/76)	8.70% (2/23)	13.21% (7/53)
Drugs	11.84% (9/76)	8.70% (2/23)	13.21% (7/53)
Retroverted uterus	7.69% (1/13)	0.00% (0/10)	33.33% (1/3)
Uterine anomalies	15.79% (12/76)	43.48% (10/23)	3.77% (2/53)
Type of uterine anomalies			
Bicornuate uterus	23.08% (3/13)	30.00% (3/10)	0.00% (0/3)
Rudimentary horn	61.54% (8/13)	60.00% (6/10)	66.67% (2/3)
Case of T-shaped uterus	7.69% (1/13)	10.00% (1/10)	0.00% (0/3)
CSP	31.58% (24/76)		45.28% (24/53)
PAS	26.32% (20/76)	13.04% (3/23)	32.08% (17/53)
Histology			
Accretism (CSP included) or abnormal intermediate trophoblast	19.74% (15/76)	13.04% (3/23)	22.64% (12/53)
Other	2.63% (2/76)	4.35% (1/23)	1.89% (1/53)
No histological anomalies	18.42% (14/76)	21.74% (5/23)	16.98% (9/53)
Unknown	59.21% (45/76)	60.87% (14/23)	58.49% (31/53)

CSP = cesarean scar pregnancy; PAS = placenta accreta spectrum; ART = assisted reproductive technology.

## Supplementary Materials

The following are available online at https://www.mdpi.com/1660-4601/17/8/2976/s1, Figure S1: methodological quality analysis, Figure S2: methodological quality analysis, Table S1: Systematic review aims, Table S2: Summary of database queries, Table S3: Maternal features and previous surgery, Table S4: Management of first trimester uterine rupture cases, Table S5: Associated factors with first trimester uterine rupture and histological findings, List S1: Included and excluded studies with reason for exclusion.
