# Supplementary material for: First Trimester Uterine Rupture: A Case Report and Literature Review
Source: Int J Environ Res Public Health. 2020 Apr 24;17(8):2976. doi: 10.3390/ijerph17082976 (PMC7215710; doi:10.3390/ijerph17082976)
Supplement: Supplementary file 1 [file ijerph-17-02976-s001.pdf]

Supplemental Table S1- Summary of the systematic review aims. The primary aim was formulated on the basis of population, exposure and outcome question.

N.	**Primary question**
1	In pregnant women affected by first-trimester spontaneous uterine rupture, what outcomes can be expected in terms of histerectomy prevalence?
	**Secondary questions**
2	In pregnant women affected by first-trimester spontaneous uterine rupture, what outcomes can be expected in addition to hysterectomy in the first trimester?
3	In pregnant women affected by first-trimester spontaneous uterine rupture, what factors are associated?
4	In pregnant women affected by first-trimester spontaneous uterine rupture, what is the management?

Supplemental Table S2- Summary of database queries.

**Database**	**Query**	**Date**	**Number of items**
PubMed	(first trimester AND uterine rupture) OR (early rupture AND gravid uterus) OR (rupture of the uterus in the first trimester) OR (pregnancy hemoperitoneum AND placenta percreta) OR (cesarean scar AND uterine rupture AND first trimester) OR (extrauterine pregnancy AND early uterine rupture)	10.04.2020	586
Scopus	(first AND trimester AND uterine AND rupture) OR early AND rupture AND gravid AND uterus) OR (rupture AND of AND the AND uterus AND in AND the AND first AND trimester) OR (pregnancy AND hemoperitoneum AND placenta AND percreta) OR (cesarean AND scar AND uterine AND rupture AND first AND trimester) OR (extrauterine AND pregnancy AND early AND uterine AND rupture) AND (LIMIT-TO (LANGUAGE , "English"))	10.04.2020	4048

Supplemental Table S3- Maternal features and previous surgery.

**Author**	**Age (years)**	**Para**	**Comorbidities**	**PS**	**Type of PS**	**Months***	**Singleton/twin pregnancy**
Hefny [25]	19	0	NK	No		NA	Singleton
Masia [46]	24	0	Turner's syndrome	Yes	D&C	NA	Singleton
Bandarian [14]	32	1-2	NK	Yes	CS + D&C	NA	Singleton
Akbas [9]	36	≥3	NK	No		NA	Singleton
Pandey [54]	30	1-2	NK	No		NA	Singleton
Singh 2013 [66]	24	0	NK	No		NA	Singleton
Shaik [62]	24	1-2	No	Yes	Myomectomy + CS	NA	Singleton
Shah [61]	39	1-2	NK	Yes	CS	NA	Singleton
Amro 1 [12]	27	1-2	NK	No		NA	Singleton
Amro 1 [12]	34	1-2	NK	No		NA	Singleton
Sultana [70]	25	1-2	NK	Yes	CS	NA	Singleton
Ijaz [27]	34	1-2	NK	Yes	D&C	NA	Singleton
Kim [37]	43	1-2	NK	Yes	CS	24	Singleton
Visariya [78]	23	1-2	NK	Yes	CS	6	Singleton
Jang [31]	27	1-2	NK	Yes	D&C	NA	Singleton
Galinski [24]	22	0	NK	No		NA	Singleton

Mahajan [43]	34	0	Fibroids	Yes	Myomectomy	36	Singleton
Dandawate [20]	39	1-2	Anemia	Yes	CS	36	Singleton
Liao [41]	29	0	No	Yes	Left salpingectomy	NA	Twins
Willmott [79]	24	0	No	No		NA	Singleton
Kapoor [35]	29	1-2	NK	No		NA	Twins
Tanyi [73]	28	≥3	No	Yes	CS + D&C	NA	Singleton
Matsuo [47]	43	1-2	Adenomyosis	Yes	CS + D&C	96	Singleton
Panayotidis [53]	32	0	No	No		NA	Singleton
Khan [36]	33	1-2	NK	Yes	CS	18	Singleton
Dabulis [19]	34	≥3	NK	Yes	CS + D&C	NA	Singleton
Ismail [28]	35	1-2	NK	Yes	CS	NA	Singleton
Tufail [76]	36	0	No	Yes	D&C	6	Singleton
Park [55]	27	1-2	NK	No		NA	Twins
Esmans [23]	41	1-2	No	Yes	D&C	NA	Singleton
Porcu [57]	36	0	NK	No		NA	Singleton
Liang [40]	34	1-2	NK	Yes	CS	36	Singleton
Singh 2000 [64]	30	≥3	No	No		NA	Singleton
Jwarah [33]	36	1-2		Yes	CS	NA	Singleton
Marcus [45]	24	1-2	NK	Yes	CS	NA	Singleton
Hamsho [10]	34	≥3	Obesity	Yes	CS	8	Singleton
DeRoux [21]	22	1-2	Sickle cell anemia	Yes	D&C	NA	Singleton
Ito [29]	19	1-2	NK	Yes	CS	72	Singleton
Ozeren [52]	32	0	NK	Yes	Myomectomy	30	Singleton
Arbab 1 [13]	28	0	Endometriosis, hyperprolactinaemia	Yes	Cornual resection	12	Singleton
Arbab 1 [13]	28	1-2	NK	Yes	Cornual resection	12	Twins
Smith [68]	34	≥3	Baltic myoclonus	Yes	CS	NA	Singleton
Biljan [16]	39	≥3	Adenomyosis	No		NA	Singleton
Abbas 2018A [8]	27	1-2	NK	Yes	CS	24	Singleton
Dibbs [22]	29	0	LES	Yes	D&C	84	Singleton
Pridjian [58]	27	0	Sarcoma, skeletal hypoplasia, delayed puberty	No		NA	Singleton
Marcellus [44]	34	1-2	Fibroids	Yes	CS	60	Singleton
Iddenden [26]	32	≥3	No	No		NA	Singleton
Jerve [32]	26	0	No	No		NA	Singleton
Lazarus [38]	39	1-2	NK	Yes	CS	7	Singleton
Bruand [17]	18	0		Yes	D&C	NA	Singleton
Saghafi [60]	34	1-2	No	Yes	CS	NA	Singleton
Takashima [72]	27	1-2	Infertility, fibroids	Yes	Myomectomy + CS	12	Singleton
Ambrogi [11]	32	1-2	NK	Yes	CS	NA	Singleton
Bechem [15]	43	0	NK	Yes	D&C	NA	Singleton
Lincenberg [42]	27	≥3	No	Yes	CS	NA	Twins
Okada [51]	34	0	Fibroids, adenomyosis	Yes	Myomectomy	7	Singleton
Sherer [63]	36	≥3	No	Yes	CS	NA	Singleton
Tola [75]	34	0	NK	No		NA	Singleton
Singh 2012 [65]	24	1-2	NK	No		NA	Singleton
Nassar [50]	26	1-2	NK	Yes	Uterine perforation + D&C	4	Singleton
Peyser [56]	35	≥3	No	Yes	D&C	NA	Singleton
Lee [39]	32	1-2	NK	No		NA	Singleton
Abbas 2018 [7]	24	1-2	NK	No		NA	Singleton
Abbas 2017 [6]	25	1-2	NK	Yes	CS	24	Singleton

Vaezi [77]	34	1-2	No	No		NA	Singleton
Cho [18]	24	0	No	No		NA	Singleton
Rouzi [59]	41	≥3	NK	Yes	CS	NA	Singleton
Mosad [49]	28	1-2	NK	Yes	CS	6	Singleton
Sujatha [69]	32	1-2	NK	Yes	CS	36	Singleton
Kabra [34]	43	≥3	NL	Yes	CS	48	Singleton
Sinha [67]	39	≥3	NK	Yes	CS + repair	24	Singleton
Miranda [48]	29	1-2	Obesity	Yes	CS	10	Singleton
Taskin [74]	34	1-2	NK	Yes	CS	NA	Singleton
Surve [71]	39	1-2	NK	Yes	CS	36	Singleton
Jain [30]	25	≥3	NK	Yes	CS	12	Singleton

* months from previous surgery
PS = previous surgery
D&C = dilation and curettage
CS = cesarean section
NK = not known

Supplemental Table 4- Management of first trimester uterine rupture cases.

Author	Diagnostic intervention	Life-saving surgery	LPS	LPT	Conversion	Defect repair	Site of rupture	Hysterectomy	Total blood loss
Hefny [25]	Yes	No	No	Yes	No	Yes	Rudimentary horn	No	2500 mL
Masia [46]	No	Yes	No	No	Yes	No	Fundus	Yes	1200 mL
Bandarian [14]	No	Yes	No	Yes	No	Yes	Previous CS	No	1500 mL
Akbas [9]	No	Yes	No	Yes	No	No	Left side	Yes	NK
Pandey [54]	No	Yes	No	Yes	No	No	Rudimentary horn	No	1000 mL
Singh 2013 [66]	Yes	No	No	Yes	No	Yes	Rudimentary horn	No	2500 mL
Shaik [62]	No	Yes	No	Yes	No	Yes	Fundus	No	2000 mL
Shah [61]	No	Yes	No	Yes	No	No	Previous CS	Yes	3000 mL
Amro 1 [12]	Yes	No	Yes	No	No	Yes	Fundus	No	2000 mL
Amro 1 [12]	Yes	No	Yes	No	No	Yes	Fundus	No	1500 mL
Sultana [70]	Yes	Yes	No	Yes	No	No	Previous CS	Yes	NK
Ijaz [27]	No	Yes	No	No	Yes	Yes	Fundus	No	1000 mL
Kim [90]	No	Yes	Yes	No	No	Yes	Left uterine horn	No	500 mL
Visariya [78]	No	Yes	No	Yes	No	Yes	Previous CS	No	1000 mL
Jang [31]	Yes	No	No	No	Yes	No	Fundus	Yes	1800 mL
Galinski [24]	No	Yes	No	Yes	No	Yes	Rudimentary horn	No	1500 mL
Mahajan [43]	Yes	No	Yes	No	No	Yes	Previous myomectomy	No	1000 mL
Dandawate [20]	Yes	No	No	No	Yes	No	Previous CS	Yes	5500 mL
Liao [41]	Yes	No	Yes	No	No	Yes	Previous section	No	0 mL
Willmott [79]	Yes	No	No	Yes	No	Yes	Fundus	No	4500 mL
Kapoor [35]	Yes	No	No	No	Yes	Yes	Rudimentary horn	No	5000 mL
Tanyi [73]	No	No	No	Yes	No	No	Anterior wall	Yes	400 mL
Matsuo [91]	No	No	No	Yes	No	Yes	Previous CS	No	240 mL
Panayotidis [92]	No	Yes	No	Yes	No	Yes	Rudimentary horn	No	3000 mL
Khan [36]	No	Yes	No	Yes	No	Yes	Previous CS	No	2000 mL
Dabulis [19]	No	Yes	No	Yes	No	No	Previous CS	Yes	3500 mL

Ismail [28]	No	No	No	No	No	No	Previous CS	No	NK
Tufail [76]	No	Yes	No	Yes	No	Yes	Rudimentary horn	No	NK
Park [55]	No	No	No	Yes	No	Yes	Fundus	No	NK
Esmans [23]	No	Yes	No	Yes	No	No	Fundus	Yes	3000 mL
Porcu [57]	Yes	No	No	No	Yes	Yes	Anterior wall	No	1500 mL
Liang [40]	No	Yes	No	Yes	No	No	Previous CS	Yes	2700 mL
Singh 2000 [64]	No	Yes	No	Yes	No	No	Fundus	Yes	1500 mL
Jwarah [33]	Yes	Yes	No	Yes	No	No	Previous CS	Yes	NK
Marcus [45]	Yes	No	No	Yes	No	No	Previous CS	Yes	NK
Hamsho [10]	No	Yes	No	Yes	No	No	Previous CS	Yes	2000 mL
DeRoux [21]	No	No	No	No	No	No	Fundus	No	7500 mL
Ito [29]	Yes	No	No	Yes	No	Yes	Previous CS	No	0 mL
Ozeren [52]	No	Yes	No	Yes	No	Yes	Previous CS	No	2000 mL
Arbab 1 [13]	No	Yes	No	Yes	No	Yes	Left side	No	severe hemorragia
Arbab 1 [13]	Yes	Yes	No	No	Yes	No	Righ side	Yes	severe hemorragia
Smith [68]	No	Yes	No	Yes	No	Yes	Fundus	No	1700 mL
Biljan [16]	No	Yes	No	Yes	No	Yes	Fundus	No	2000 mL
Abbas 2018A [8]	Yes	Yes	No	Yes	No	Yes	Previous CS	No	1500 mL
Dibbs [22]	Yes	No	No	Yes	No	Yes	Fundus	No	2000 mL
Pridjian [58]	No	Yes	No	Yes	No	No	Anterior wall	Yes	1000 mL
Marcellus [44]	No	Yes	No	Yes	No	No	Previous CS	Yes	2000 mL
Iddenden [26]	No	Yes	No	Yes	No	Yes	Fundus	No	3000 mL
Jerve [32]	No	Yes	No	Yes	No	Yes	Lower segment	No	NK
Lazarus [38]	No	Yes	No	Yes	No	No	Previous CS	Yes	NK
Bruand [17]	Yes	Yes	Yes	No	Yes	No	Right uterine horn	No	1000 mL
Saghafi [60]	Yes	No	No	Yes	No	Yes	Previous CS	No	100 mL
Takashima [72]	No	Yes	No	Yes	No	No	Previous CS	Yes	1300 mL
Ambrogi [11]	No	No	No	No	Yes	No	Posterior wall	Yes	NK
Bechem [15]	No	No	No	Yes	No	Yes	Fundus	No	NK
Lincenberg [42]	Yes	No	No	No	Yes	Yes	Previous CS	No	900 mL
Okada [51]	Yes	No	No	Yes	No	Yes	Fundus	No	860 mL
Sherer [63]	Yes	No	No	No	Yes	Yes	Previous CS	No	NK
Tola [75]	No	Yes	No	Yes	No	Yes	Rudimentary horn	No	1000 mL
Singh 2012 [65]	Yes	Yes	No	Yes	No	Yes	Rudimentary horn fundus	No	2500 mL
Nassar [50]	Yes	No	No	Yes	No	Yes	Fundus	No	1500 mL
Peyser [56]	No	Yes	No	Yes	No	No	Righ side	Yes	NK
Lee [39]	Yes	No	Yes	No	No	No	Fundus	Yes	1000 mL
Abbas 2018 [7]	Yes	No	No	Yes	No	No	Posterior wall	No	2500 mL
Abbas 2017 [6]	No	Yes	No	Yes	No	Yes	Previous CS	No	1000 mL
Vaezi [77]	Yes	No	No	Yes	No	Yes	Posterior wall	No	2000 mL
Cho [18]	No	Yes	No	Yes	No	Yes	Fundus	No	2500 mL
Rouzi [59]	No	Yes	No	Yes	No	No	Previous CS	Yes	NK
Mosad [49]	No	Yes	No	Yes	No	Yes	Previous CS	No	NK
Sujatha [69]	No	Yes	No	Yes	No	Yes	Previous CS	No	1800 mL
Kabra [34]	No	Yes	No	Yes	No	Yes	Previous CS	No	1500 mL
Sinha [67]	No	Yes	No	Yes	No	Yes	Fundus	No	1000 mL

Miranda [48]	Yes	No	No	Yes	No	Yes	Previous CS	No	NK
Taskin [74]	Yes	No	No	Yes	No	Yes	Previous CS	No	NK
Surve [71]	No	Yes	No	Yes	No	Yes	Previous CS	No	2000 mL
Jain [30]	No	Yes	No	Yes	No	Yes	Previous CS	No	2000 mL

LPS = laparoscopy
LPT = laparotomy
NK = not known

Supplemental Table S5- Associated factors with first trimester uterine rupture and histological findings after surgical intervention.

Author	ART	Drugs	Uterine anomalies	Type of uterine anomalies	CSP	PAS	Histology
Hefny [25]	No	No	Yes	Bicornuate uterus	No	No	NK
Masia [46]	Yes	No	No		No	Yes	NK
Bandarian [14]	No	No	No		Yes	No	NK
Akbas [9]	No	No	No		No	No	NK
Pandey [54]	No	No	Yes	Rudimentary horn	No	No	NK
Singh 2013 [66]	No	No	Yes	Bicornuate uterus	No	No	No histopathological abnormalities
Shaik [62]	No	No	No		Yes	NK	NK
Shah [61]	No	Yes	No		Yes	Yes	NK
Amro 1 [12]	No	No	No		No	No	NK
Amro 1 [12]	Yes	No	No		No	No	NK
Sultana [70]	No	No	No		Yes	NK	NK
Ijaz [27]	No	No	No		No	No	No histopathological abnormalities
Kim [90]	No	Yes	No		No	No	No histopathological abnormalities
Visariya [78]	No	No	No		Yes	NK	NK
Jang [31]	No	No	No		No	No	Accretism
Galinski [24]	No	No	Yes	Rudimentary horn	No	No	No histopathological abnormalities
Mahajan [43]	No	No	No		No	No	NK
Dandawate [20]	No	No	No		Yes	Yes	Accretism
Liao [41]	Yes	No	No		No	No	No histopathological abnormalities
Willmott [79]	No	Yes	No		No	No	No histopathological abnormalities
Kapoor [35]	No	No	Yes	Rudimentary-horn	No	No	Rudimentary horn
Tanyi [73]	No	No	No		No	Yes	Accretism
Matsuo [91]	No	No	No		No	No	NK
Panayotidis [92]	No	No	Yes	Rudimentary-horn	No	No	No histopathological abnormalities
Khan [36]	No	Yes	No		NK	NK	NK
Dabulis [19]	No	No	No		Yes	Yes	Accretism
Ismail [28]	No	Yes	No		No	No	No histopathological abnormalities
Tufail [76]	No	No	Yes	Rudimentary horn	No	No	No histopathological abnormalities
Park [55]	No	No	No		No	No	NK
Esmans [23]	No	No	No		No	Yes	Accretism
Porcu [57]	No	No	Yes	T-shaped and hypopalstic uterus	No	No	NK
Liang [40]	No	No	No		Yes	Yes	Accretism
Singh 2000 [64]	No	No	Yes	Rudimentary-horn	No	No	No histopathological abnormalities
Jwarah [33]	No	Yes	No		NK	NK	NK
Marcus [45]	No	No	No		Yes	Yes	NK
Hamsho [10]	No	No	No		Yes	NK	NK
DeRoux [21]	No	No	No		No	Yes	Accretism
Ito [29]	Yes	No	No		Yes	NK	NK
Ozeren [52]	No	No	No		No	No	NK
Arbab 1 [13]	Yes	No	No		No	No	No histopathological abnormalities
Arbab 1 [13]	Yes	No	No		No	Yes	Accretism

Smith [68]	No	No	No		No	Yes	NK
Biljan [16]	No	No	No		No	No	NK
Abbas 2018A [8]	No	No	No		Yes	Yes	NK
Dibbs [22]	No	No	No		No	No	Abnormal intermediate trophoblast
Pridjian [58]	No	No	NK		No	Yes	Accretism
Marcellus [44]	No	No	No		No	Yes	Accretism
Iddenden [26]	No	No	No		No	No	NK
Jerve [32]	No	Yes	No		No	No	NK
Lazarus [38]	No	No	No		Yes	NK	NK
Bruand [17]	No	No	Yes	Rudimentary horn	NK	NK	NK
Saghafi [60]	No	No	No		No	No	No histopathological abnormalities
Takashima [72]	Yes	No	No		Yes	NK	NK
Ambrogi [11]	No	No	No		No	Yes	Accretism
Bechem [15]	No	No	No		No	No	NK
Lincenberg [42]	No	No	No		Yes	Yes	NK
Okada [51]	Yes	No	No		No	No	NK
Sherer [63]	No	No	No		Yes	Yes	Choriocarcinoma
Tola [75]	No	No	Yes	Bicornuate uterus	No	No	NK
Singh 2012 [65]	No	No	Yes	Bicornuate uterus, rudimentary horn	No	NK	NK
Nassar [50]	No	No	No		No	No	No histopathological abnormalities
Peyser [56]	No	Yes	No		No	No	NK
Lee [39]	No	No	No		No	Yes	Accretism
Abbas 2018 [7]	No	No	No		No	NK	NK
Abbas 2017 [6]	No	No	No		Yes	NK	NK
Vaezi [77]	No	No	No		No	NK	NK
Cho [18]	Yes	No	No		No	Yes	Accretism
Rouzi [59]	No	Yes	No		Yes	NK	NK
Mosad [49]	No	No	No		Yes	Yes	No histopathological abnormalities
Sujatha [69]	No	No	No		Yes	No	NK
Kabra [34]	No	No	No		Yes	NK	CSP
Sinha [67]	No	No	No		No	No	NK
Miranda [48]	No	No	No		Yes	NK	NK
Taskin [74]	No	No	No		NK	NK	NK
Surve [71]	No	No	No		Yes	NK	NK
Jain [30]	No	No	No		Yes	NK	NK

CSP = cesarean scar pregnancy
PAS = placenta accreta spectrum
RVF = retroverse uterus
ART = assisted reproductive technology.

Supplemental Figure S1- Summary diagram of the methodological quality analysis of the included studies (part 1).Color legend: green (+) item present (high quality), yellow (?) not clear, and red (-) item missing (low quality).

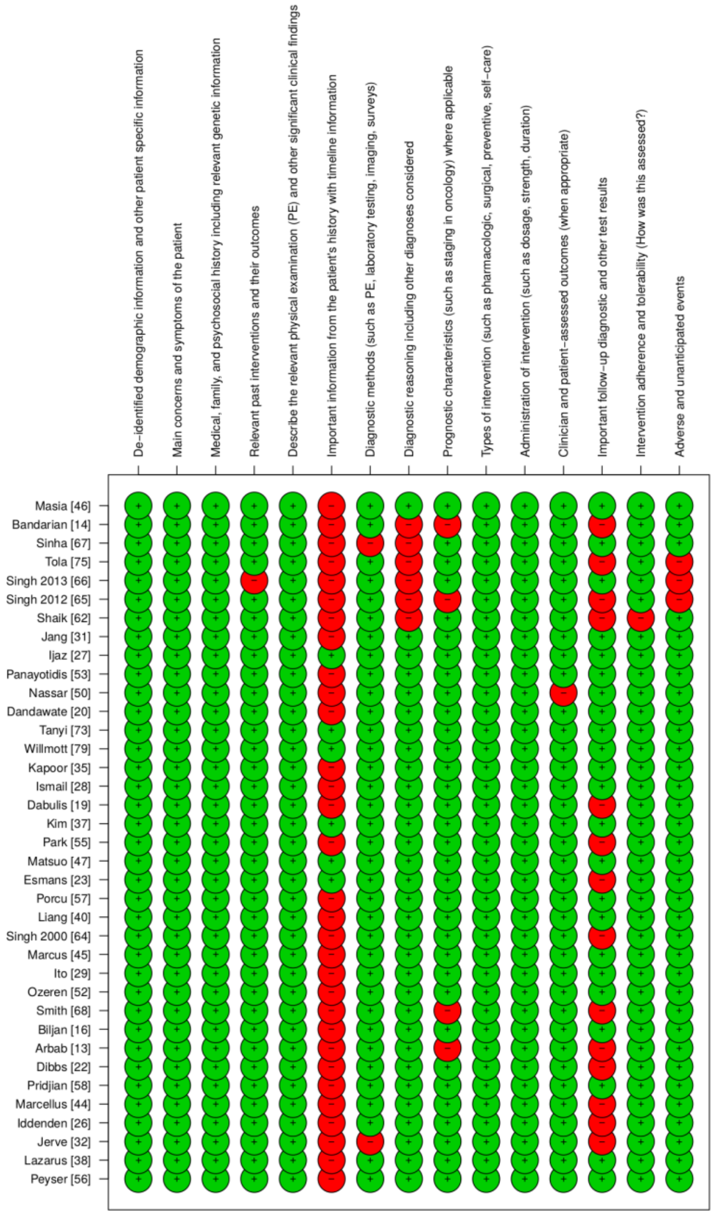

Supplemental Figure 2- Summary diagram of the methodological quality analysis of the included studies (part 2).Color legend: green (+) item present (high quality), yellow (?) not clear, and red (-) item missing (low quality).

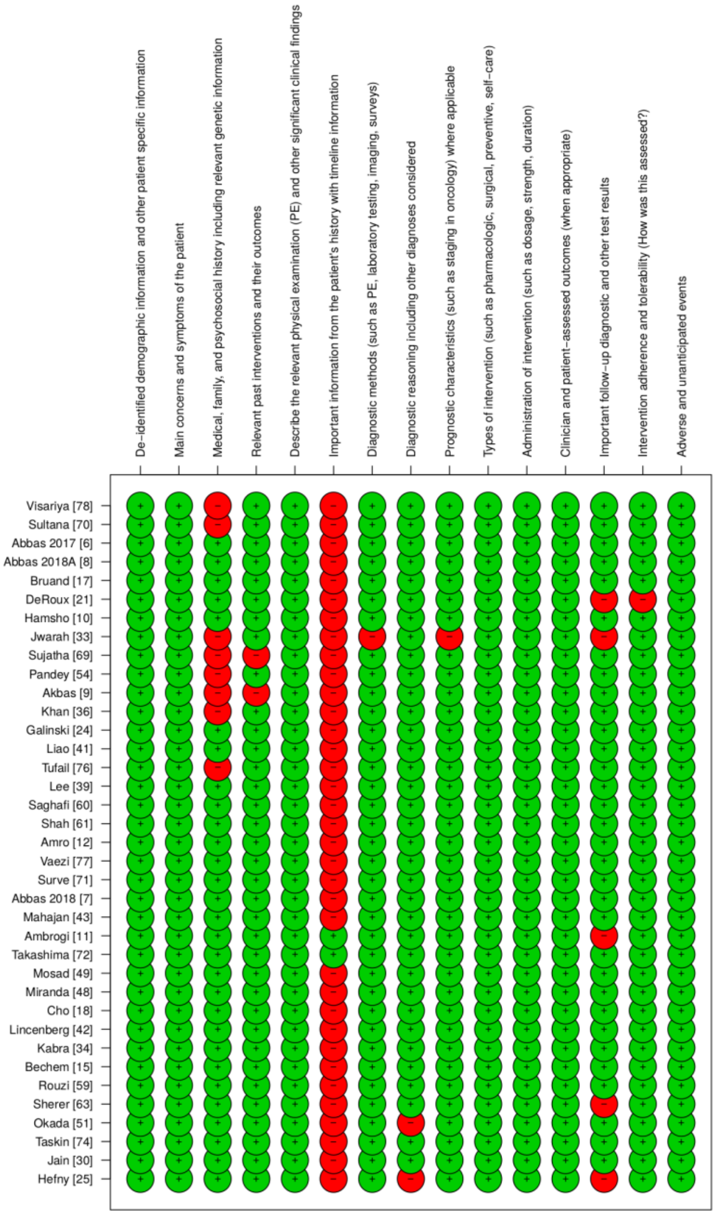
De−identified demographic information and other patient specific information
Main concerns and symptoms of the patient
Medical, family, and psychosocial history including relevant genetic information
Relevant past interventions and their outcomes
Describe the relevant physical examination (PE) and other significant clinical findings
Important information from the patient's history with timeline information
Diagnostic methods (such as PE, laboratory testing, imaging, surveys)
Diagnostic reasoning including other diagnoses considered
Prognostic characteristics (such as staging in oncology) where applicable
Types of intervention (such as pharmacologic, surgical, preventive, self−care)
Administration of intervention (such as dosage, strength, duration)
Clinician and patient−assessed outcomes (when appropriate)
Important follow−up diagnostic and other test results
Intervention adherence and tolerability (How was this assessed?)
Adverse and unanticipated events
Visariya [78]
Sultana [70]
Abbas 2017 [6]
Abbas 2018A [8]
Bruand [17]
DeRoux [21]
Hamsho [10]
Jwarah [33]
Sujatha [69]
Pandey [54]
Akbas [9]
Khan [36]
Galinski [24]
Liao [41]
Tufail [76]
Lee [39]
Saghafi [60]
Shah [61]
Amro [12]
Vaezi [77]
Surve [71]
Abbas 2018 [7]
Mahajan [43]
Ambrogi [11]
Takashima [72]
Mosad [49]
Miranda [48]
Cho [18]
Lincenberg [42]
Kabra [34]
Bechem [15]
Rouzi [59]
Sherer [63]
Okada [51]
Taskin [74]
Jain [30]
Hefny [25]

Supplemental List S1

INCLUDED STUDIES

Studies that fulfilled the requirements.

References

[1] Abbas AM, Ali SS, Michael A, et al. Caesarean Scar Ectopic Pregnancy Complicated by Uterine Rupture at 10 Weeks Gestation. J Gynecol Surg. 2017;33:261–263.

[2] Abbas AM, Hussein RS, Ali MN, et al. Spontaneous First Trimester Posterior Uterine Rupture in a Multiparous Woman with Scarred Uterus: A Case Report. Middle East Fertility Society Journal. 2018;23:81–83.

[3] Abbas AM, Michael A, Ali SS, et al. Placenta Percreta Presenting with Marked Hemoperitoneum in the First Trimester of Pregnancy: A Case Report. Middle East Fertil Soc J. 2018;23:251–253.

[4] Akbaş M, Ömeroğlu İ, Bİrge Ö. Spontaneous Uterine Rupture with Retroperitoneal Hematoma in the First Trimester: Case Report. Turkiye Klinikleri J Gynecol Obst. 2015;25:205–208.

[5] Alloub AH M. Spontaneous Rupture of a 13 Week Gravid Scarred Uterus. Journal of Obstetrics and Gynaecology. 1999;19:316–317.

[6] Ambrogi G, Ambrogi G, Marchi AA. Placenta Percreta and Uterine Rupture in the First Trimester of Pregnancy. Case Rep Obstet Gynecol. 2018;2018:6842892.

[7] Amro B, Lotfi G. Spontaneous Rupture of an Unscarred Uterus in Early Pregnancy: A Rare but Life-Threatening Emergency. BMJ Case Rep. 2019;12:e228493.

[8] Arbab F, Boulieu D, Bied V, et al. Uterine Rupture in First or Second Trimester of Pregnancy after In-Vitro Fertilization and Embryo Transfer. Human Reproduction. 1996;11:1120–1122.

[9] Bandarian M, Bandarian F. Spontaneous Rupture of the Uterus during the 1st Trimester of Pregnancy. J Obstet Gynaecol. 2015;35:199–200.

[10] Bechem E, Leopold D, Ako TW. Small Bowel Exteriorisation after Uterine Perforation from Manual Vacuum Aspiration for Abortion in a Young Cameroonian: A Case Report. Pan Afr Med J. 2016;25.

[11] Biljan MM, Cushing K, McDicken IW, et al. Spontaneous Uterine Rupture in the First Trimester of Pregnancy. Journal of Obstetrics and Gynaecology. 1996;16:174–175.

[12] Bruand M, Thubert T, Winer N, et al. Rupture of Non-Communicating Rudimentary Horn of Uterus at 12 Weeks' Gestation. Cureus. 2020;.

[13] Cho MK, Ryu HK, Kim CH. Placenta Percreta-Induced Uterine Rupture at 7th Week of Pregnancy After In Vitro Fertilization in a Primigravida Woman: Case Report. J Emerg Med. 2017;53:126–129.

[14] Dabulis SA, McGuirk TD. An Unusual Case of Hemoperitoneum: Uterine Rupture at 9 Weeks Gestational Age. The Journal of Emergency Medicine. 2007;33:285–287.

[15] Dandawate B, Carpenter T. Caesarean Scar Pregnancy Presenting as Anaemia. Journal of Obstetrics and Gynaecology. 2009;29:772–773.

[16] deRoux SJ, Prendergast NC, Adsay NV. Spontaneous Uterine Rupture with Fatal Hemoperitoneum Due to Placenta Accreta Percreta: A Case Report and Review of the Literature. Int J Gynecol Pathol. 1999;18:82–86.

[17] Dibbs KI, Ball RH, Huettner PC. Spontaneous Uterine Rupture and Hemoperitoneum in the First Trimester. Am J Perinatol. 1995;12:439–441.

[18] Esmans A, Gerris J, Corthout E, et al. Placenta Percreta Causing Rupture of an Unscarred Uterus at the End of the First Trimester of Pregnancy: Case Report. Hum Reprod. 2004;19:2401–2403.

[19] Galinski M, Petrovic T, Rodrigues A, et al. Out-of-Hospital Diagnosis of a Ruptured Ectopic Pregnancy: Myometrial Embryo Implantation, an Exceptional Diagnosis. Prehospital Emergency Care. 2010;14:496–498.

[20] Hefny AF, Kunhivalappil FT, Nambiar R, et al. A Rare Case of First-Trimester Ruptured Bicornuate Uterus in a Primigravida. International Journal of Surgery Case Reports. 2015;14:98–100.

[21] Iddenden DA, Nuttall ID. Early Spontaneous Rupture of the Gravid Uterus. Am J Obstet Gynecol. 1983; 147:971–972.

[22] Ijaz S, Mahendru A, Sanderson D. Spontaneous Uterine Rupture during the 1st Trimester: A Rare but Life-Threatening Emergency. Journal of Obstetrics and Gynaecology. 2011;31:772–772.

[23] Ismail SIMF, Toon PG. First Trimester Rupture of Previous Caesarean Section Scar. Journal of Obstetrics and Gynaecology. 2007;27:202–204.

[24] Ito M, Nawa T, Mikamo H, et al. Lower Segment Uterine Rupture Related to Early Pregnancy by in Vitro Fertilization and Embryo Transfer after a Previous Cesarean Delivery. J Med. 1998;29:85–91.

[25] Jain S, Chaudhary S, Jain N, et al. Ruptured Caesarean Scar Ectopic Pregnancy: A Rare Case Report. Int J Reprod Contracept Obstet Gynecol. 2015;3011–3012.

[26] Jang DG, Lee GSR, Yoon JH, et al. Placenta Percreta-Induced Uterine Rupture Diagnosed by Laparoscopy in the First Trimester. Int J Med Sci. 2011;8:424–427.

[27] Jerve F, Fylling P, Stenby S. Rupture of the Uterus Following Treatment with 16-16-Dimethyl E 2 Prostaglandin Vagitories. Prostaglandins. 1979;17:121–123.

[28] Jwarah E, Greenhalf JO. Rupture of the Uterus after 800 Micrograms Misoprostol given Vaginally for Termination of Pregnancy. BJOG. 2000;107:807.

[29] Kabra SL, Laul P, Godha Z, et al. Case Series: Spontaneous Rupture of Uterus in Early Pregnancy. J Obstet Gynecol India. 2016;66:710–713.

[30] Kapoor S, Gardner FJE, de Chazal R, et al. Ruptured Rudimentary Horn and TRAP Syndrome. Journal of Obstetrics and Gynaecology. 2008;28:358–359.

[31] Khan S, Alison G. Uterine Rupture at 8 Weeks' Gestation Following 600 *M*g of Oral Misoprostol for Management of Delayed Miscarriage. Journal of Obstetrics and Gynaecology. 2007;27:869–870.

[32] Kim JO, Han JY, Choi JS, et al. Oral Misoprostol and Uterine Rupture in the First Trimester of Pregnancy: A Case Report. Reproductive Toxicology. 2005;20:575–577.

[33] Lazarus EJ. Early Rupture of the Gravid Uterus. American Journal of Obstetrics and Gynecology. 1978; 132:224.

[34] Lee F, Zahn K, Knittel AK, et al. Laparoscopic Hysterectomy to Manage Uterine Rupture Due to Placenta Percreta in the First Trimester: A Case Report. Case Rep Womens Health. 2020;25:e00165.

[35] Liang HS, Jeng CJ, Sheen TC, et al. First-Trimester Uterine Rupture from a Placenta Percreta. A Case Report. J Reprod Med. 2003;48:474–478.

[36] Liao CY, Ding DC. Repair of Uterine Rupture in Twin Gestation after Laparoscopic Cornual Resection. Journal of Minimally Invasive Gynecology. 2009;16:493–495.

[37] Lincenberg KR, Behrman ER, Bembry JS, et al. Uterine Rupture with Cesarean Scar Heterotopic Pregnancy with Survival of the Intrauterine Twin. Case Rep Obstet Gynecol. 2016;2016:6832094.

[38] Mahajan N, Moretti ML, Lakhi NA. Spontaneous Early First and Second Trimester Uterine Rupture Following Robotic-Assisted Myomectomy. Journal of Obstetrics and Gynaecology. 2019;39:278–280.

[39] Marcellus M, Jenkins DM, Keohane C. Intra Abdominal Rupture of First Trimester Cervical Pregnancy. IJMS. 1989;158:20–21.

[40] Marcus S, Cheng E, Goff B. Extrauterine Pregnancy Resulting from Early Uterine Rupture. Obstet Gynecol. 1999;94:804–805.

[41] Masia F, Zoric L, Ripart-Neveu S, et al. Spontaneous Uterine Rupture at 14 Weeks Gestation during a Pregnancy Consecutive to an Oocyte Donation in a Woman with Turner's Syndrome. Anaesthesia Critical Care & Pain Medicine. 2015;34:101–103.

[42] Matsuo K, Shimoya K, Shinkai T, et al. Uterine Rupture of Cesarean Scar Related to Spontaneous Abortion in the First Trimester. J Obstet Gynaecol Res. 2004;30:34–36.

[43] Miranda A, Castro L, José Rocha M, et al. Uterine Rupture in Early Pregnancy. International Journal of Pregnancy & Child Birth. 2017;2.

[44] Mosad A, Altraigey A. Scar Pregnancy and Spontaneous Rupture Uterus - a Case Report. Ginekol Pol. 2017;88:698–699.

[45] Nassar AH, Charara I, Nawfal AK, et al. Ectopic Pregnancy in a Uterine Perforation Site. American Journal of Obstetrics and Gynecology. 2009;201:e15–e16.

[46] Okada Y, Hasegawa J, Mimura T, et al. Uterine Rupture at 10 Weeks of Gestation after Laparoscopic Myomectomy. J Med Ultrasonics. 2016;43:133–136.

[47] Ozeren M, Ulusoy M, Uyanik E. First-Trimester Spontaneous Uterine Rupture after Traditional Myomectomy: Case Report. Isr J Med Sci. 1997;33:752–753.

[48] Panayotidis C, Abdel-Fattah M, Leggott M. Rupture of Rudimentary Uterine Horn of a Unicornuate Uterus at 15 Weeks' Gestation. Journal of Obstetrics and Gynaecology. 2004;24:323–324.

[49] Pandey D, Tripathi BN, Mishra P. Rudimentary Horn Pregnancy: A Rare First Trimester Acute Presentation. Int J Womens Health Reprod Sci. 2015;3:115–117.

[50] Park YJ, Ryu KY, Lee JI, et al. Spontaneous Uterine Rupture in the First Trimester: A Case Report. J Korean Med Sci. 2005;20:1079–1081.

[51] Peyser MR, Toaff R. Rupture of Uterus in the First Trimester Caused by High-Conentration Oxytocin Drip. Obstet Gynecol. 1972;40:371–372.

[52] Porcu G, Courbière B, Sakr R, et al. Spontaneous Rupture of a First-Trimester Gravid Uterus in a Woman Exposed to Diethylstilbestrol in Utero. A Case Report. J Reprod Med. 2003;48:744–746.

[53] Pridjian G, Rich NE, Montag AG. Pregnancy Hemoperitoneum and Placenta Percreta in a Patientwith Previous Pelvic Irradiation and Ovarian Failure. American Journal of Obstetrics and Gynecology. 1990; 162:1205–1206.

[54] Rouzi AA, Almarzouki A, Tallab F, et al. Medical Management of Early Pregnancy Failure with Misoprostol with Rupture of the Cesarean Section Scar Pregnancy. Clin Exp Obstet Gynecol. 2017;44:477–479.

[55] Saghafi N, Maleki A, Ayati S, et al. First Trimester Uterine Rupture, a Rare but Life-Threatening Event: A Case Report. Iranian Journal of Medical Sciences. 2019;44.

[56] Shah P, Manandhar R, Thapa M, et al. Ruptured Cesarean Scar Pregnancy: A Case Report. J Nepal Med Assoc. 2019;57.

[57] Shaikh S, Shaikh NB, Channa S, et al. First Trimester Uterine Rupture Due to Scar Ectopic Pregnancy. Med Channel. 2012;19:68–70.

[58] Sherer DM, Dalloul M, Cho Y, et al. Spontaneous First-Trimester Perforation of the Uterus Following Cesarean Scar Pregnancy Choriocarcinoma: CSP Choriocarcinoma. Ultrasound Obstet Gynecol. 2016; 47:519–521.

[59] Singh A, Jain S. Spontaneous Rupture of Unscarred Uterus in Early Pregnancy: - A Rare Entity. Acta Obstet Gynecol Scand. 2000;79:431–432.

[60] Singh K, Soni A, Rana S. Ruptured Ectopic Pregnancy in Caesarean Section Scar: A Case Report. Case Rep Obstet Gynecol. 2012;2012:106892.

[61] Singh N, Singh U, Verma ML. Ruptured Bicornuate Uterus Mimicking Ectopic Pregnancy: A Case Report. J Obstet Gynaecol Res. 2013;39:364–366.

[62] Sinha P, Agrawal N. Spontaneous Uterine Rupture in First Trimester of Pregnancy. Int J Reprod Contracept Obstet Gynecol. 2014;831–832.

[63] Smith L, Mueller P. Abdominal Pain and Hemoperitoneum in the Gravid Patient: A Case Report of Placenta Percreta. The American Journal of Emergency Medicine. 1996;14:45–47.

[64] Sujatha B, Bharatnur S, Virmani S, et al. Ruptured CaesarianScar Ectopic Pregnancy. Online J Health Allied Scs. 2017;16:14.

[65] Sultana R, Islam S, Nurjahan. Caesarean Scar Pregnancy - A Rare Case Report. Bangladesh J Obstet Gynaecol. 2012;27:83–86.

[66] Surve M, Pawar S, Panigrahi PP. A Case Report ofFirst In Mester Spontaneous Uterine Scar Rupture. mmj. 2017;1:26–28.

[67] Takashima A, Takeshita N, Kinoshita T. A Case of Scarred Uterine Rupture at 11 Weeks of Gestation Having a Uterine Scar Places Induced by in Vitro Fertilization-Embryo Transfer. Clin Pract. 2018;8:1038.

[68] Tanyi JL, Coleman NM, Johnston ND, et al. Placenta Percreta at 7th Week of Pregnancy in a Woman with Previous Caesarean Section. Journal of Obstetrics and Gynaecology. 2008;28:338–340.

[69] Taskin MI, Adali E. Spontaneous Uterine Rupture in the First Trimester with Missed Fetus. J Cases Obstet Gynecol. 2015;2:97–99.

[70] Tola EN. First Trimester Spontaneous Uterine Rupture in a Young Woman with Uterine Anomaly. Case Rep Obstet Gynecol. 2014;2014:967386.

[71] Tufail A, Hashmi HA. Ruptured Ectopic Pregnancy in Rudimentary Horn of the Uterus. J Coll Physicians Surg Pak. 2007;17:105–106.

[72] Vaezi M. Unexpected Rupture of Unscarred Uterus at 12 Weeks of Pregnancy: A Case Report and Literature Review. IJWHR. 2017;5:339–341.

[73] Visariya N, Purandare CN, Bandukwalla V, et al. First Trimester Uterine Rupture Previous Lower Segment Cesarean Scar. J Obstet Gynecol India. 2011;61:88–89.

[74] Willmott FJ, Scherf C, Ford SM, et al. Rupture of Uterus in the First Trimester during Medical Termination of Pregnancy for Exomphalos Using Mifepristone/Misoprostol. BJOG. 2008;115:1575–1577.

EXCLUDED STUDIES

No uterine rupture [1–25]

Gestational age equal or greather 14 weeks [26–67]

Extra-uterine pregnancy [68, 69]

Other reasons (non-English literarure, duplicate publication, or non reporting clinical data) [70–91]

References

[1] Putra AD, Savitri QM. Cesarean-Scar Ectopic Pregnancy: A Rare Case. J Gynecol Surg. 2019;35:272–275.

[2] Rodpenpear N, Laosooksathit W, Phasipol P, et al. Cesarean Scar Pregnancy: A Case Report and Literature Review. J Med Assoc Thai. 2019;102:105.

[3] Majangara R, Madziyire MG, Verenga C, et al. Cesarean Section Scar Ectopic Pregnancy - a Management Conundrum: A Case Report. J Med Case Rep. 2019;13:137.

[4] Odgers HL, Taylor RAM, Balendran J, et al. Rupture of a Caesarean Scar Ectopic Pregnancy: A Case Report. Case Rep Womens Health. 2019;22:e00120.

[5] Glenn TL, Bembry J, Findley AD, et al. Cesarean Scar Ectopic Pregnancy: Current Management Strategies. Obstet Gynecol Surv. 2018;73:293–302.

[6] a Hamid H, Zulida R, Norhafizah M. Massive Haemorrhage Secondary to Placenta Percreta in the First Trimester: A Case Report. Clin Exp Obstet Gynecol. 2015;42:101–103.

[7] Shaamash AH, Houshimi WM, El-kanzi EmM, et al. Abortion Hysterectomy at 11weeks' Gestation Due to Undiagnosed Placenta Accreta (PA): A Case Report and a Mini Review of Literatures. Middle East Fertil Soc J. 2014;19:147–152.

[8] Ergenoglu AM, Yeniel AO, Yildirim N, et al. Early Diagnosis of Rudimentary Horn Pregnancy and Its Excision by Laparoscopy: Case Report. J Turk Soc Obstet Gynecol. 2012;9:77–79.

[9] Nawfal AK, Blacker CM, Strickler RC, et al. Laparoscopic Management of Pregnancy in a Patient with Uterus Didelphys, Obstructed Hemivagina, and Ipsilateral Renal Agenesis. J Minim Invasive Gynecol. 2011 May-Jun;18:381–385.

[10] Colomé C, Cusidó MT, Hereter L, et al. Conservative Treatment by Endoscopy of a Cesarean Scar Pregnancy: Two Case Reports. Clin Exp Obstet Gynecol. 2009;36:126–129.

[11] Kadan Y, Romano S. Rudimentary Horn Pregnancy Diagnosed by Ultrasound and Treated by Laparoscopy–a Case Report and Review of the Literature. J Minim Invasive Gynecol. 2008 Sep-Oct; 15:527–530.

[12] Iyibozkurt AC, Topuz S, Gungor F, et al. Conservative Treatment of an Early Ectopic Pregnancy in a Cesarean Scar with Systemic Methotrexate–Case Report. Clin Exp Obstet Gynecol. 2008;35:73–75.

[13] Holland MG, Bienstock JL. Recurrent Ectopic Pregnancy in a Cesarean Scar. Obstet Gynecol. 2008; 111:541–545.

[14] Taori K, Saha BK, Shah D, et al. Sonographic Diagnosis of Uncomplicated First-Trimester Pregnancy in the Rudimentary Horn of a Unicornuate Uterus. J Clin Ultrasound. 2008;36:45–47.

[15] Ozkan S, Calişkan E, Ozeren S, et al. Three-Dimensional Ultrasonographic Diagnosis and Hysteroscopic Management of a Viable Cesarean Scar Ectopic Pregnancy. J Obstet Gynaecol Res. 2007;33:873–877.

[16] Rajan S. Ectopic Pregnancy Within a Cesarean Section Scar. J Diagn Med Sonogr. 2006;22:395–398.

[17] Ko HS, Lee Y, Lee HJ, et al. Sonographic and MR Findings in 2 Cases of Intramural Pregnancy Treated Conservatively. J Clin Ultrasound. 2006;34:356–360.

[18] Chang CY, Wu MT, Shih JC, et al. Preservation of Uterine Integrity via Transarterial Embolization under Postoperative Massive Vaginal Bleeding Due to Cesarean Scar Pregnancy. Taiwan J Obstet Gynecol. 2006; 45:183–187.

[19] Ross R, Lindheim SR, Olive DL, et al. Cornual Gestation: A Systematic Literature Review and Two Case Reports of a Novel Treatment Regimen. J Minim Invasive Gynecol. 2006 Jan-Feb;13:74–78.

[20] Lee GSR, Hur SY, Kown I, et al. Diagnosis of Early Intramural Ectopic Pregnancy. J Clin Ultrasound. 2005; 33:190–192.

[21] Graesslin O, Dedecker F, Quereux C, et al. Conservative Treatment of Ectopic Pregnancy in a Cesarean Scar. Obstet Gynecol. 2005;105:869–871.

[22] Einenkel J, Stumpp P, Kösling S, et al. A Misdiagnosed Case of Caesarean Scar Pregnancy. Arch Gynecol Obstet. 2005;271:178–181.

[23] Vagg D, Arsala L, Kathurusinghe S, et al. Intramural Ectopic Pregnancy Following Myomectomy. J Investig Med High Impact Case Rep. 2018 Jan-Dec;6:2324709618790605.

[24] Hitzerd E, Bogers H, Kianmanesh Rad NA, et al. A Viable Caesarean Scar Pregnancy in a Woman Using a Levonorgestrel-Releasing Intrauterine Device: A Case Report. Eur J Contracept Reprod Health Care. 2018; 23:161–163.

[25] Abdelkader MA, Fouad R, Gebril AH, et al. Caesarean Scar Pregnancy: Hysterotomy Is Rapid and Safe Management Option. Arch Gynecol Obstet. 2014;290:381–383.

[26] Sun Y, Huang J, Kong HF. Spontaneous Rupture of Unscarred Uterus in the Third Trimester after in Vitro Fertilization-Embryo Transfer Because of Bilateral Salpingectomy: A Case Report. Medicine (Baltimore). 2019; 98:e18182.

[27] Hua Z, Wu M. Spontaneous Rupture of the Uterus Following Salpingectomy: A Case Report and Literature Review. J Int Med Res. 2019;47:5328–5336.

[28] Enebe JT, Ofor IJ, Okafor II. Placenta Percreta Causing Spontaneous Uterine Rupture and Intrauterine Fetal Death in an Unscared Uterus: A Case Report. Int J Surg Case Rep. 2019;65:65–68.

[29] Nitzsche B, Dwiggins M, Catt S. Uterine Rupture in a Primigravid Patient with an Unscarred Bicornuate Uterus at Term. Case Rep Womens Health. 2017;15:1–2.

[30] Yildirim D, Turkgeldi LS, Tekiner N, et al. A Case of Rudimentary Horn Pregnancy Diagnosed after Failed Attempts at Pregnancy Termination. Niger J Clin Pract. 2017;20:111–114.

[31] Ahmed A, Nanda A, Gupta V, et al. An Unusual Case of Twin Pregnancy Associated with Rudimentary Horn Rupture. J South Asian Fed Obstet Gynaecol. 2017;9:129–130.

[32] Basak SK, Begum R, Haque J. Asymptomatic Rupture of Term Non-Communicating Rudimentary Horn of Uterus: A Case Report. Bangladesh J Obstet Gynaecol. 2015;30:53–55.

[33] Shirazi M, Shaar-Baf FR, Moosavi SA. Rupture of an Unscarred Uterus in a Nullipar Pregnant Women with a History of Curettage: Case Report. Tehran Univ Med J. 2015;73:138–142.

[34] Alam IP, Mahbuba, Forhad QE. Ruptured Rudimentary Horn Pregnancy: Two Case Reports. Bangladesh J Obstet Gynaecol. 2014;29:116–119.

[35] Kulkarni K, Ajmera S. Pregnancy in Rudimentary Horn of Uterus. Indian J Med Sci. 2013 Jan-Feb;67:45–47.

[36] IQBAL R, KHURSHID S, INTASAR A. Ruptured Ectopic Pregnancy in the Rudimentary Horn of Uterus at 14 Weeks-A Case Report at Fatima Memorial Hospital. 2012;.

[37] Uccella S, Cromi A, Bogani G, et al. Spontaneous Prelabor Uterine Rupture in a Primigravida: A Case Report and Review of the Literature. Am J Obstet Gynecol. 2011;205:e6–e8.

[38] Dahiya K, Duhan N, Nanda S. Rudimentary Horn Pregnancy: Series of Four Cases and Review of the Literature. J Gynecol Surg. 2011;27:139–141.

[39] Kawthalkar AS, Gawande MS, Jain SH, et al. Rare Case of Live Birth in a Ruptured Rudimentary Horn Pregnancy. J Obstet Gynaecol Res. 2011;37:1169–1172.

[40] Hung FY, Wang PT, Weng SL, et al. Placenta Percreta Presenting as a Pinhole Uterine Rupture and Acute Abdomen. Taiwan J Obstet Gynecol. 2010;49:115–116.

[41] Soliman N, Babar SA. Spontaneous Rupture of the Uterus Secondary to Placenta Percreta with Conservation of the Uterus. J Obstet Gynaecol. 2010;30:517–518.

[42] Chopra S, Keepanasseril A, Rohilla M, et al. Obstetric Morbidity and the Diagnostic Dilemma in Pregnancy in Rudimentary Horn: Retrospective Analysis. Arch Gynecol Obstet. 2009;280:907–910.

[43] Low YS, Adams T, Clement-Jones M. Uterine Rupture during the Mid-Trimester Management of Intrauterine Fetal Death. J Obstet Gynaecol. 2009;29:443.

[44] Ramphal SR, Moodley J. Antepartum Uterine Rupture in Previous Caesarean Sections Presenting as Advanced Extrauterine Pregnancies: Lessons Learnt. Eur J Obstet Gynecol Reprod Biol. 2009;143:3–8.

[45] Zahumensky J, Sottner O, Brtnicka H, et al. Pregnancy and Delivery After Ectopic Pregnancy in a Caesarean Section Scar. Geburtshilfe Frauenheilkd. 2008;68:389–392.

[46] Matsuo K, Scanlon JT, Atlas RO, et al. Staircase Sign: A Newly Described Uterine Contraction Pattern Seen in Rupture of Unscarred Gravid Uterus. J Obstet Gynaecol Res. 2008;34:100–104.

[47] Naim NM, Ahmad S, Siraj HH, et al. Advanced Abdominal Pregnancy Resulting from Late Uterine Rupture. Obstet Gynecol. 2008;111:502–504.

[48] Panayotidis C, Prabhu S. Management of Un-Ruptured Pregnancy in a Rudimentary Horn of Unicornuate Uterus at 14 Weeks Gestation. Gynecol Surg. 2007;4:281–284.

[49] Kaczmarczyk M, Sparén P, Terry P, et al. Risk Factors for Uterine Rupture and Neonatal Consequences of Uterine Rupture: A Population-Based Study of Successive Pregnancies in Sweden. BJOG. 2007; 114:1208–1214.

[50] Dasari P. Case Report: Pre-Rupture Ultrasound Diagnosis of Rudimentary Horn Pregnancy. 2007;.

[51] Teunissen KK, Lopriore E, Nijman RGW, et al. Silent Uterine Rupture, an Unusual Cause of Anhydramnios. Am J Obstet Gynecol. 2007;196:e8–9.

[52] Thomas EO, Gordon J, Smith-Thomas S, et al. Diffuse Uterine Leiomyomatosis with Uterine Rupture and Benign Metastatic Lesions of the Bone. Obstet Gynecol. 2007;109:528–530.

[53] Koul I, Wakhloo A, bala Gupta S. Catastrophic Haemorrhage after the Rupture of Rudimentary Horn Pregnancy. JK-Pract. 2006;13:212–213.

[54] Wang YL, Su TH. Obstetric Uterine Rupture of the Unscarred Uterus: A Twenty-Year Clinical Analysis. Gynecol Obstet Invest. 2006;62:131–135.

[55] Karnik A, Shah JR, Pungavkar SA, et al. Conversion of Intra-Uterine Pregnancy into Abdominal Pregnancy Due to Ruptured Uterus: Preoperative Sonographic and MRI Diagnosis:. J Womens Imaging. 2005;7:199–204.

[56] Honig A, Rieger L, Thanner F, et al. Placenta Percreta with Subsequent Uterine Rupture at 15 Weeks of Gestation after Two Previous Cesarean Sections. J Obstet Gynaecol Res. 2005;31:439–443.

[57] Api M, Api O. CATASTROPHIC HEMORRHAGE AFTER RUPTURE OF RUDIMENTARY HORN PREGNANCY WHICH ONCE MISDIAGNOSED AS UTERUS BICORNIS. Pak J Med Sci. 2005;21:217–9.

[58] Hlibczuk V. Spontaneous Uterine Rupture as an Unusual Cause of Abdominal Pain in the Early Second Trimester of Pregnancy. J Emerg Med. 2004;27:143–145.

[59] Oral B, Güney M, Ozsoy M, et al. Placenta Accreta Associated with a Ruptured Pregnant Rudimentary Uterine Horn. Case Report and Review of the Literature. Arch Gynecol Obstet. 2001;265:100–102.

[60] Nkemayim DC, Hammadeh ME, Hippach M, et al. Uterine Rupture in Pregnancy Subsequent to Previous Laparoscopic Electromyolysis. Case Report and Review of the Literature. Arch Gynecol Obstet. 2000; 264:154–156.

[61] Suner S, Jagminas L, Peipert JF, et al. Fatal Spontaneous Rupture of a Gravid Uterus: Case Report and Literature Review of Uterine Rupture. J Emerg Med. 1996 Mar-Apr;14:181–185.

[62] Passini Júnior R, Knobel R, Barini R, et al. Placenta Percreta with Silent Rupture of the Uterus. Sao Paulo Med J. 1996 Sep-Oct;114:1270–1273.

[63] Bevan JR, Marley NJ, Ozumba EN. Uterine Rupture, Placenta Percreta and Curettage in Early Pregnancy. Case Report. Br J Obstet Gynaecol. 1985;92:642–644.

[64] Martínez-Garza PA, Robles-Landa LPA, Roca-Cabrera M, et al. Spontaneous Uterine Rupture: Report of Two Cases. Cir Cir. 2012 Jan-Feb;80:81–85.

[65] Hong SC, Lau MSK, Yam PKL. Ectopic Pregnancy in Previous Caesarean Section Scar. Singapore Med J. 2011;52:e115–117.

[66] Oktem O, Gökaslan H, Durmusoglu F. Spontaneous Uterine Rupture in Pregnancy 8 Years after Laparoscopic Myomectomy. J Am Assoc Gynecol Laparosc. 2001;8:618–621.

[67] Cava EF, Russell WM. Intramural Pregnancy with Uterine Rupture: A Case Report. Am J Obstet Gynecol. 1978;131:214–216.

[68] Rodrigues Â, Neves AR, Castro MG, et al. Successful Management of a Rudimentary Uterine Horn Ectopic Pregnancy by Combining Methotrexate and Surgery: A Case Report. Case Rep Womens Health. 2019; 24:e00158.

[69] Gibson KR, Horne AW. Ruptured Heterotopic Pregnancy: An Unusual Presentation of an Uncommon Clinical Problem. Case Rep. 2012;2012:bcr2012007423–bcr2012007423.

[70] Deldar K, Saleh Seyedein S, Lotfalizadeh M, et al. Report of Two Cases of Missed Uterus Rupture after Normal Vaginal Delivery without Previous Scar. IJOGI. 2019;21.

[71] Paquette K, Markey S, Roberge S, et al. First and Third Trimester Uterine Scar Thickness in Women With Previous Caesarean: A Prospective Comparative Study. J Obstet Gynaecol Can. 2019;41:59–63.

[72] Ishida H, Takashima A, Nagaoka M, et al. Uterine Rupture Due to Placenta Percreta in the First Trimester of a Pregnancy Subsequent to a Transverse Uterine Fundal Cesarean Section: A Case Report: Rupture after Transverse Fundal CS. J Obstet Gynaecol Res. 2018;44:1832–1835.

[73] Pontis A, Prasciolu C, Litta P, et al. Uterine Rupture in Pregnancy: Two Case Reports and Review of Literature. Clin Exp Obstet Gynecol. 2016;43:304–309.

[74] Dorairajan G. Ruptured Uterus. Springer Singapore, Singapore. 2017.

[75] Kim DJ, Welch M, Kendall JL. A Case of Cesarean Scar Ectopic: A Rare but Important Form of Ectopic Pregnancy. Crit Ultrasound J. 2011;3:55–57.

[76] Jiang T, Liu G, Huang L, et al. Methotrexate Therapy Followed by Suction Curettage Followed by Foley Tamponade for Caesarean Scar Pregnancy. Eur J Obstet Gynecol Reprod Biol. 2011;156:209–211.

[77] Al-Zirqi I, Stray-Pedersen B, Forsén L, et al. Uterine Rupture after Previous Caesarean Section. BJOG. 2010;117:809–820.

[78] Hidar S, Benregaya L, Elabed M, et al. All First Trimester Uterine Ruptures Caused by Scar Implantation? Med Hypotheses. 2010;74:616.

[79] Sliutz G, Sanani R, Spängler-Wierrani B, et al. First Trimester Uterine Rupture and Scar Pregnancy. Med Hypotheses. 2009;73:326–327.

[80] Nicks BA, Fitch MT, Manthey DE. A Case of Intrauterine Molar Pregnancy with Coexistent Ectopic Pregnancy. J Emerg Med. 2009;36:246–249.

[81] Muppala H, Najia SK, Clarke FR. Current Evidence in the Management of Previous Caesarean Section: Clinical Review. Eur Clin Obstet Gynaecol. 2007;3:67–80.

[82] Walsh CA, Baxi LV. Rupture of the Primigravid Uterus: A Review of the Literature. Obstet Gynecol Surv. 2007;62:327–334; quiz 353–354.

[83] Ash A, Smith A, Maxwell D. Caesarean Scar Pregnancy. BJOG. 2007;114:253–263.

[84] Chopra S, Suri V, Aggarwal N. Rudimentary Horn Pregnancy: Prerupture Diagnosis and Management. Indian J Med Sci. 2007;61:28–29.

[85] Murphy DJ. Uterine Rupture. Curr Opin Obstet Gynecol. 2006;18:135–140.

[86] Hidar S, Jerbi M, Khaïri H. Uterine Rupture after 400 Micrograms Misoprostol Oral Administration in the First Trimester of Pregnancy. Reprod Toxicol. 2006;21:223; author reply 224.

[87] Han JY, Nava-Ocampo AA. Oral Misoprostol and Uterine Rupture in the First Trimester of Pregnancy. Reprod Toxicol. 2006;21:224.

[88] Klemm P, Koehler C, Mangler M, et al. Laparoscopic and Vaginal Repair of Uterine Scar Dehiscence Following Cesarean Section as Detected by Ultrasound. J Perinat Med. 2005;33:324–331.

[89] Hofmeyr GJ, Say L, Gülmezoglu AM. WHO Systematic Review of Maternal Mortality and Morbidity: The Prevalence of Uterine Rupture. BJOG. 2005;112:1221–1228.

[90] Jayasinghe Y, Rane A, Stalewski H, et al. The Presentation and Early Diagnosis of the Rudimentary Uterine Horn. Obstet Gynecol. 2005;105:1456–1467.

[91] Axelsson H, Winbland B. Dislocated IUD and Intrauterine Ectopic Pregnancy with Uterine Rupture. Obstet Gynecol. 1976;47:365–366.
